# Artificial intelligence-based computer-aided system for knee osteoarthritis assessment increases experienced orthopaedic surgeons’ agreement rate and accuracy

**DOI:** 10.1007/s00167-022-07220-y

**Published:** 2022-11-11

**Authors:** Maria Anna Smolle, Christoph Goetz, Dietmar Maurer, Ines Vielgut, Michael Novak, Gerhard Zier, Andreas Leithner, Stefan Nehrer, Tiago Paixao, Richard Ljuhar, Patrick Sadoghi

**Affiliations:** 1 Department of Orthopaedics and Trauma, Medical University of Graz, Auenbruggerplatz 5, 8036 Graz, Austria; 2 ImageBiopsy Lab, Zehetnergasse 6/2/2, 1140 Vienna, Austria; 3 Department of Orthopaedics, Landeskrankenhaus Südsteiermark, Standort Radkersburg, Dr.-Schwaiger-Straße 1, 8490 Bad Radkersburg, Austria; 4 Diagnosehaus, Hans-Sachs-Gasse 10-12, 1180 Wien, Austria; 5 Danube University Krems, Dr. Karl-Dorrek Straße 30, 3500 Krems, Austria

**Keywords:** Knee osteoarthritis, Artificial intelligence, Computer aided detection, Reader study

## Abstract

**Purpose:**

The aims of this study were to (1) analyze the impact of an artificial intelligence (AI)-based computer system on the accuracy and agreement rate of board-certified orthopaedic surgeons (= senior readers) to detect X-ray features indicative of knee OA in comparison to unaided assessment and (2) compare the results to those of senior residents (= junior readers).

**Methods:**

One hundred and twenty-four unilateral knee X-rays from the OAI study were analyzed regarding Kellgren–Lawrence grade, joint space narrowing (JSN), sclerosis and osteophyte OARSI grade by computerized methods. Images were rated for these parameters by three senior readers using two modalities: plain X-ray (*unaided*) and X-ray presented alongside reports from a computer-assisted detection system (*aided*). After exclusion of nine images with incomplete annotation, intraclass correlations between readers were calculated for both modalities among 115 images, and reader performance was compared to ground truth (OAI consensus). Accuracy, sensitivity and specificity were also calculated and the results were compared to those from a previous study on junior readers.

**Results:**

With the aided modality, senior reader agreement rates for KL grade (2.0-fold), sclerosis (1.42-fold), JSN (1.37-fold) and osteophyte OARSI grades (3.33-fold) improved significantly. Reader specificity and accuracy increased significantly for all features when using the aided modality compared to the gold standard. On the other hand, sensitivity only increased for OA diagnosis, whereas it decreased (without statistical significance) for all other features. With aided analysis, senior readers reached similar agreement and accuracy rates as junior readers, with both surpassing AI performance.

**Conclusion:**

The introduction of AI-based computer-aided assessment systems can increase the agreement rate and overall accuracy for knee OA diagnosis among board-certified orthopaedic surgeons. Thus, use of this software may improve the standard of care for knee OA detection and diagnosis in the future.

**Level of evidence:**

Level II.

## Introduction

Characterized by functional disability and chronic pain, knee osteoarthritis (OA) accounts for approximately one-fifth of the OA of all joints [1]. OA may be diagnosed clinically or radiologically, though symptoms may be present years prior to the first appearance of X-ray signs indicative of OA [2, 3]. The most frequently used classification for knee OA is the *Kellgren Lawrence* (KL) scale, which differentiates five stages (0–4) of OA severity [4]. However, the KL scale is criticized for its assumption of linear OA progression [5] as well as its differing interpretations leading to aberrant classification of especially low-grade knee OA [6]. Therefore, *Osteoarthritis Research Society International* (OARSI) has developed an OA classification system based on an atlas with exemplary X-rays of distinct features [7].

While magnetic resonance imaging (MRI) has gained importance in the diagnosis of musculoskeletal pathologies, the advantages of plain X-ray over MRI include their prevalent availability and cost efficiency. However, early-stage OA signs are invisible on plain X-rays, as cartilage degeneration cannot be directly assessed, and OA constitutes a three-dimensional problem [8]. This is reflected by fair to moderate interobserver reliability for knee OA assessment using X-rays alone, with measured quadratic kappa values between 0.56 and 0.67 [9–11]. To overcome these issues, different solutions, including novel quantitative grading methods and automatic knee X-ray assessment tools, have been proposed [6, 12–14]. Currently, artificial intelligence (AI) and deep learning are used in medical image classification related to the musculoskeletal system [15–17].

In this study, the authors aimed to characterize two aspects of the impact of a novel, AI-based image annotation tool with regard to changes in the radiological judgement of knee OA [18]. First, we analyzed the intra- and interobserver reliability of board-certified orthopaedic surgeons (herein termed senior readers) regarding knee OA grade assessment using either AI-annotated or plain X-rays. Second, we compared the outcome of senior readers to that of senior residents (termed junior readers) with aided analysis in terms of agreement rate and overall performance.

## Methods

Three board-certified orthopaedic surgeons (= senior readers) from a single hospital rated X-ray images, and readings with and without AI aid were compared to a gold standard (OAI consensus). The findings of a similar previous study [19] involving senior residents (= junior readers) were used as a comparator for senior reader performance.

### Data

In the present study, plain knee X-rays were acquired from a publicly available dataset by *Osteoarthritis Initiative *(*OAI)* [20]. From this dataset, 124 knee X-rays (size comparable to previous study in this field [21]) were semirandomly selected using a selection probability proportional to the frequency of KL grades across the visits baseline, as previously described [19]. Thereby, a uniform distribution of KL grades was ensured in the sample set. The images used in the present study and training data of the AI were drawn from OAI but segregated by the patient level to avoid biasing AI performance due to overfitting. Overfitting implies that an AI model has been trained in a way that the learned methodology is only applicable to the training set but not to another independent dataset [22]. A few additional images from the OAI dataset, outside of the study set, were randomly chosen for training of the readers on the user interface of the study’s annotation tool (see below).

Table 1 depicts the distribution of KL and OARSI grades of the final cohort (*n* = 115; 9 images with incomplete annotation by readers excluded), as reported by consensus readings of the OAI study (i.e. ground truth). Table 2 contains the patient demographics of the final cohort stratified by sex. All knee X-rays from the OAI study followed a “fixed flexion” protocol, with standing X-rays in posterior-anterior (PA) projection with feet externally rotated by 10° and knees flexed to 20–30° (until the knees and thighs touch the vertical X-ray table anteriorly) [23–25].

**Table 1:** Distribution of KL and OARSI grades in the study population, as reported by the gold standard consensus readings of *Osteoarthritis Initiative* [20] (OAI; *n* = 115)

	KL grade	OARSI grade		
		Osteophytes	JSN	Sclerosis
0	24 (20.9)	34 (29.6)	46 (40.0)	40 (34.8)
1	18 (15.7)	35 (30.4)	28 (24.3)	24 (20.9)
2	34 (29.5)	17 (14.8)	20 (17.4)	23 (20.0)
3	24 (20.9)	29 (25.2)	21 (18.3)	28 (24.3)
4	15 (13.0)	N/A	N/A	N/A

*JSN* joint space narrowing, *KL* Kellgren–Lawrence

**Table 2:** Population demographics of the individuals analyzed in this study (*n* = 115)

	Total (*n* = 115; %)	Female (*n* = 60; %)	Male (*n* = 55; %)
Age groups			
45–49 years	5 (4.4)	0 (0.0)	5 (9.1)
50–59 years	32 (27.8)	18 (30.0)	14 (25.5)
60–69 years	36 (31.3)	22 (36.7)	14 (25.5)
70–79 years	35 (30.4)	17 (28.3)	18 (32.7)
80–89 years	7 (6.1)	3 (5.0)	4 (7.2)
Ethnicity			
Asian	1 (0.9)	0 (0.0)	1 (1.8)
Black or African American	24 (20.8)	15 (25.0)	9 (16.4)
Other Non-White	1 (0.9)	1 (1.7)	0 (0.0)
White or Caucasian	89 (77.4)	44 (73.3)	45 (81.8)
BMI			
20–25	24 (20.9)	12 (20.0)	12 (21.8)
25–30	45 (39.1)	20 (33.3)	25 (45.5)
30–35	31 (27.0)	17 (28.3)	14 (25.5)
35–40	15 (13.0)	11 (18.3)	4 (7.3)

### Knee osteoarthritis labeling assistant

KOALA (*Knee OsteoArthritis Labeling Assistant*) is a software providing both metric assessments of anterior–posterior (AP) or PA knee X-rays and proposals for clinical OA grade. These proposals should aid clinicians in assessing the degree of knee OA in adult patients or their risk of developing it. This is enabled by standardized quantitative measurements of morphological features (i.e. joint space width [JSW] and joint space area [JSA]) on AP or PA knee X-rays. The AI software subsequently provides numerical results together with graphical overlays on X-rays showing measurement points. Furthermore, OA severity is assessed with the AI software by proposing the following grades (the higher, the more severe) to the clinician/rater: maximum OARSI grade for sclerosis, joint space narrowing (JSN), osteophytes (each between 0 and 3), and KL grade (between 0 and 4). Grading proposals as well as metric assessments are summarized in a report that can be viewed on any DICOM viewer workstation approved by the FDA.

The AI software applied in this study is based on several CNNs trained on large datasets of over 20,000 individual knee X-rays. It combines several low- and high-level modules, with the low-level modules being responsible for knee joint detection and landmarking. Subsequently, information is transferred to the high-level modules responsible for joint segmentation and measurement of KL, JSW, and OARSI grades, as previously described [19].

### Labelling process

**Fig. 1:**
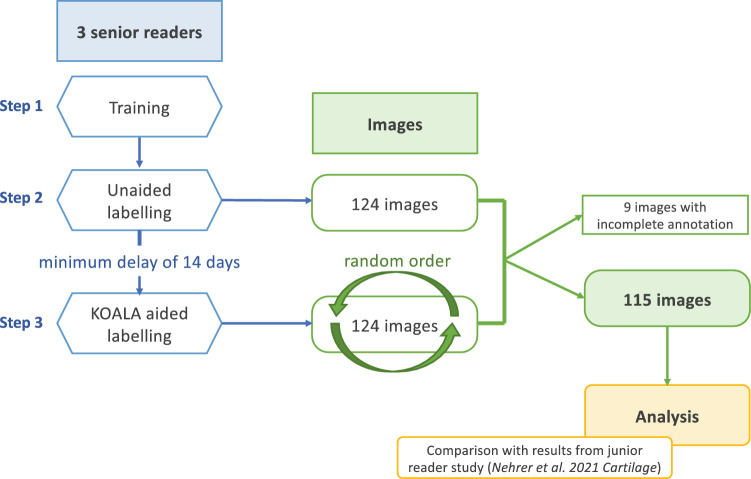
Flow chart of the study’s labelling process

The labelling process was divided into three steps (Fig. 1). First, three senior readers were trained on the structure of the AI software report, OARSI grading system [7], labelling process and platform used (i.e. Labelbox, a data labelling tool designed for machine learning procedures [26]. Second, readers assessed—unaided (i.e. without AI annotations)—124 plain knee X-rays and defined KL grade (0–4), osteophytes (0–3), sclerosis (0–3), and JSN (0–3) by completing a list. The readers were able to work remotely at their preferred time and allowed to interrupt and resume labelling at any time, without time restrictions for labelling individual images or the entire dataset. However, the time it took readers to label each image, as well as the time of the entire labelling process, was ascertained. Third, after a minimum of 2 weeks after the second step had been completed, the same 124 knee X-rays were relabelled by the readers, with images provided at random order (to avoid creating observer bias; Fig. 1). At this point, however, each image was supplemented with the AI software’s report together with a binary score of whether OA was present on the X-ray.

As mentioned above, complete data by annotators for all modalities were not available for nine images; therefore, these were excluded from further analysis, resulting in a total subject count of 115 and a dropout rate of 7.3%.

### Statistical analysis

#### Agreement rates

A two-way random, single score model intraclass correlation coefficient (ICC) was used to assess agreement rates between readers for items evaluated (i.e. presence of OA, osteophytes, sclerosis, KL grade, JSN) [27] when compared to the OAI consensus. As proposed by *Shrout and Fleiss*, 95% confidence intervals (CIs) were calculated [27]. Standard errors of the mean were estimated for ICCs by resampling observations with 1000 bootstraps. Via the *z* score method, the statistical significance of the difference between the unaided and aided labelling was determined. A *p* value of < 0.01 was considered statistically significant.

#### Accuracy measures

The performance of the readers was assessed by accuracy, sensitivity and specificity. True positives (TP), true negatives (TN), false-positives (FP) and false negatives (FN) were calculated for each measure of the readers against the readings from the OAI study. In particular, the ability to detect any abnormality (KL grade > 0) or OA (KL grade > 1), JSN (> 0), sclerosis (> 0) or severe sclerosis (> 1) and the presence of osteophytes (> 0) was assessed. Normal approximation to binomial proportional intervals was used to estimate standard errors as well as CIs for sensitivity, specificity, and accuracy.

#### Receiver operating characteristic curves

As the AI software used not only provides recommendations regarding the presence and severity of OA but also a confidence score on the recommendation made, a receiver operating characteristic (ROC) curve was plotted to visualize the effect of the AI software’s application on reader performance with regard to changes in TP rates (TPR) and FP rates (FPR).

## Results

### Agreement between readers in unaided and aided labelling

**Fig. 2:**
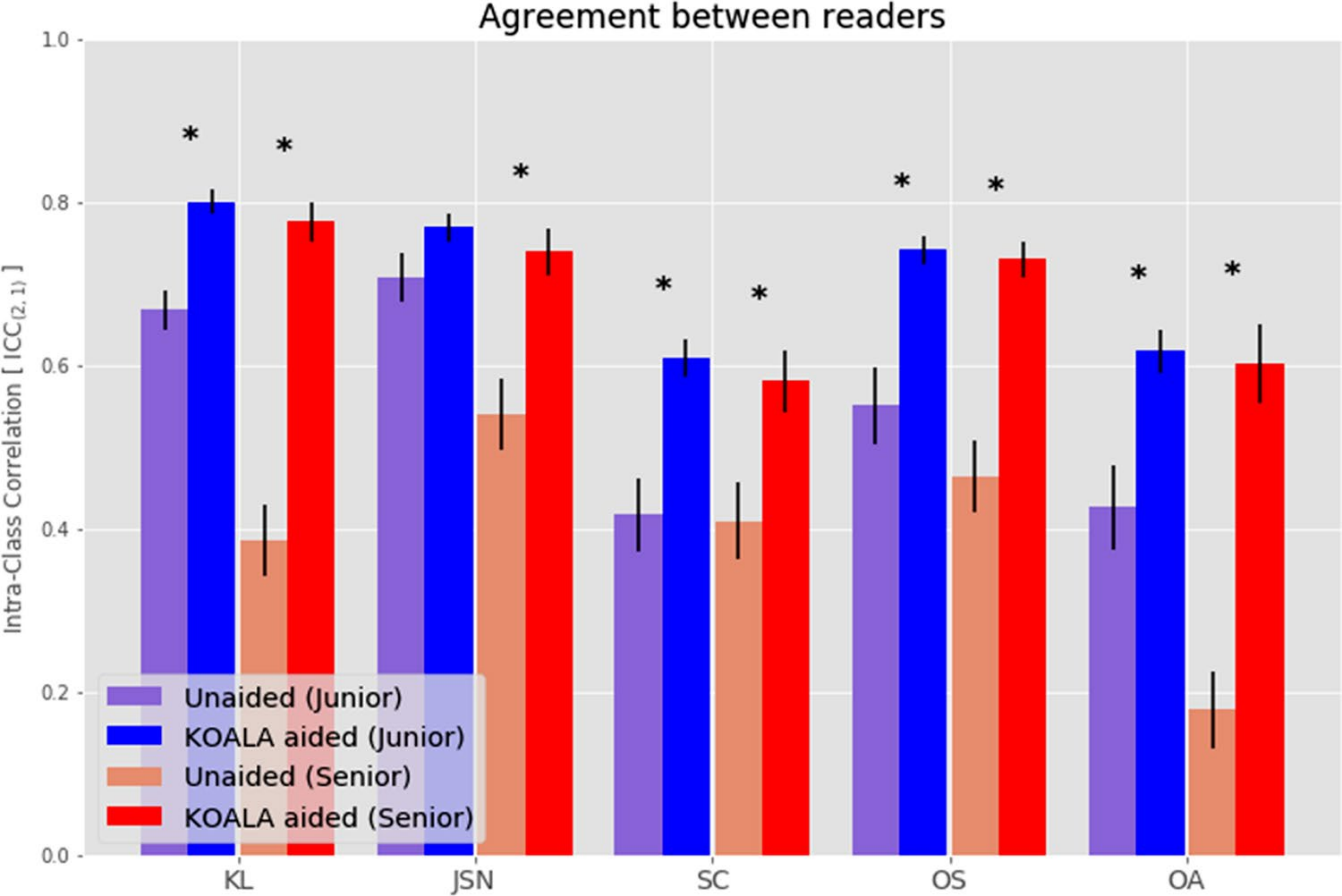
Agreement rates between junior readers (blue) and senior readers (red) for the unaided (lighter) and aided (darker) modalities. Error bars denote standard errors of the ICC. Stars indicate statistically significant differences between the unaided and aided modalities, with a *p* value of < 0.01 considered statistically significant. JSN junior was the only nonsignificant result, with a *p* value of 0.125. Horizontal lines denote the thresholds separating poor, fair, good and excellent agreement, as defined by Cicchetti et al. [28]. The values for junior readers already published by Nehrer et al. [19]. OA was defined as KL > 1. *KL* Kellgren–Lawrence, *JSN* joint space narrowing, *SC* sclerosis, *OS* osteophyte, *OA* osteoarthritis)

Agreement rates for different measures (i.e. KL, presence of OA, JSN, sclerosis, osteophytes) between readers were calculated separately for unaided and aided labelling. Agreement rates for senior readers improved with aided labelling for all scores assessed (Table 3, Fig. 2). In detail, agreement rates increased twofold, 1.37-fold, 1.42-fold, 1.59-fold and 3.33-fold for KL grade, JSN, sclerosis, osteophytes and OA diagnosis, respectively (Table 3). When using the agreement classification proposed by Cicchetti [28], improvements from unaided to aided labelling were observed for all measures, except for sclerosis (Table 3).

**Table 3:** Agreement rates (ICCs) for junior and senior readers, by modality, together with the Cicchetti agreement classification [28]

Score	Junior			Senior		
	Unaided	Aided	Change (unaided-aided)	Unaided	Aided	Change (unaided–aided)
KL						
ICC	0.67 (0.58–0.74)	0.82 (0.76–0.86)	× 1.22	0.39 (0.06–0.62)	0.78 (0.70–0.84)	× 2.0
Cicchetti	Good	Excellent		Poor	Excellent	
JSN						
ICC	0.71 (0.62–0.78)	0.76 (0.67–0.83)	× 1.07	0.54 (0.34–0.68)	0.74 (0.67–0.80)	× 1.37
Cicchetti	Good	Excellent		Fair	Good	
Sclerosis						
ICC	0.42 (0.30–0.53)	0.60 (0.50–0.69)	× 1.43	0.41 (0.16–0.60)	0.58 (0.46–0.68)	× 1.42
Cicchetti	Fair	Good		Fair	Fair	
Osteo-phytes						
ICC	0.55 (0.25–0.73)	0.75 (0.65–0.83)	× 1.36	0.46 (0.23–0.64)	0.73 (0.66–0.79)	× 1.59
Cicchetti	Fair	Excellent		Fair	Good	
OA						
ICC	0.43 (0.32–0.54)	0.60 (0.50–0.69)	× 1.40	0.18 (0.06–0.30)	0.60 (0.51–0.69)	× 3.33
Cicchetti	Fair	Good		Poor	Good	

The values for junior readers have already been published by *Nehrer *et al*.* [19]. Significant changes (defined as *p* value < 0.01) are highlighted in grey shading

In general, agreement rates for KL grade, JSN, and OA diagnosis with unaided labelling were higher between junior readers than between senior readers (Table 3, Fig. 2). Notably, when using aided labelling, ICCs for the junior and senior readers were comparable. Consequently, less pronounced improvements in ICC from unaided to aided labelling were found with junior readers (Table 3).

#### Senior reader performance in unaided and aided labelling

**Fig. 3:**
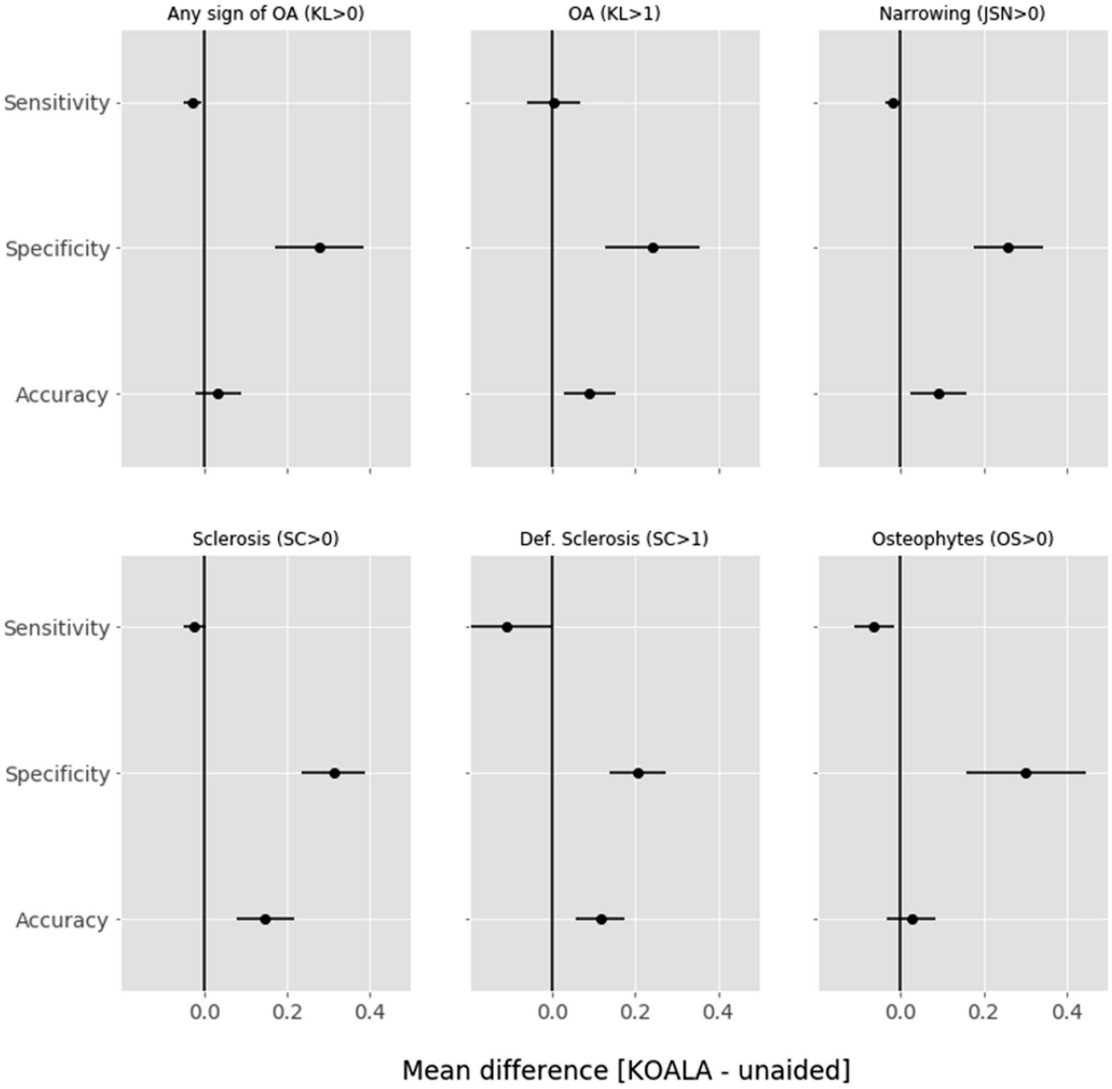
Mean differences between senior reader AI software-aided and unaided labelling in sensitivity, specificity and accuracy for KL > 0, KL > 1, JSN > 0, sclerosis OARSI grade > 0, sclerosis OARSI grade > 1 and osteophyte OARSI grade > 0. Values to the right of the vertical line at 0 are improvements by the use of AI software. Error bars signify 95% confidence intervals. *KL* Kellgren–Lawrence, *JSN* joint space narrowing, *SC* sclerosis, *OS* osteophyte, *OA* osteoarthritis

The accuracy of senior readers significantly improved with aided labelling for all measures (Fig. 3). Sensitivity only increased for OA diagnosis (i.e. KL grade > 1) while decreasing—without statistical significance—for all other scores. On the other hand, all measures showed a significant increase in specificity, indicating a decrease in overdiagnosis upon AI-aided labelling compared to the OAI ground truth (Fig. 3, Table 4).

**Table 4:** Accuracy, sensitivity and specificity of senior readers with unaided and aided modalities. The increase from unaided to aided labelling is highlighted in green, and the decrease is highlighted in red

Modality	Accuracy	Sensitivity	Specificity
KL (> 0)			
Unaided	0.80 (0.76–0.84)	0.97 (0.95–0.99)	0.10 (0.03–0.18)
Aided	0.86 (0.82 – 0.89)	0.97 (0.94–0.99)	0.40 (0.29–0.52)
JSN (> 0)			
Unaided	0.65 (0.60 – 0.70)	0.97 (0.95–0.99)	0.11 (0.06–0.17)
Aided	0.76 (0.72–0.80)	0.97 (0.95–0.99)	0.41 (0.32 – 0.49)
Sclerosis (> 0)			
Unaided	0.54 (0.49–0.59)	0.97 (0.95–0.99)	0.11 (0.07–0.15)
Aided	0.60 (0.55–0.65)	0.97 (0.94–0.99)	0.24 (0.18–0.30)
Severe sclerosis (> 1)			
Unaided	0.72 (0.67–0.76)	0.80 (0.73–0.88)	0.68 (0.62–0.74)
Aided	0.88 (0.84–0.91)	0.77 (0.68–0.84)	0.92 (0.89–0.96)
Osteophytes (> 0)			
Unaided	0.76 (0.71–0.80)	0.84 (0.79–0.88)	0.50 (0.39–0.61)
Aided	0.84 (0.80–0.88)	0.88 (0.83–0.91)	0.73 (0.63–0.82)
OA (KL > 1)			
Unaided	0.76 (0.72–0.81)	0.83 (0.77–0.87)	0.65 (0.58–0.73)
Aided	0.82 (0.78–0.85)	0.78 (0.73–0.83)	0.88 (0.82–0.93)

#### Individual reader performance

**Fig. 4:**
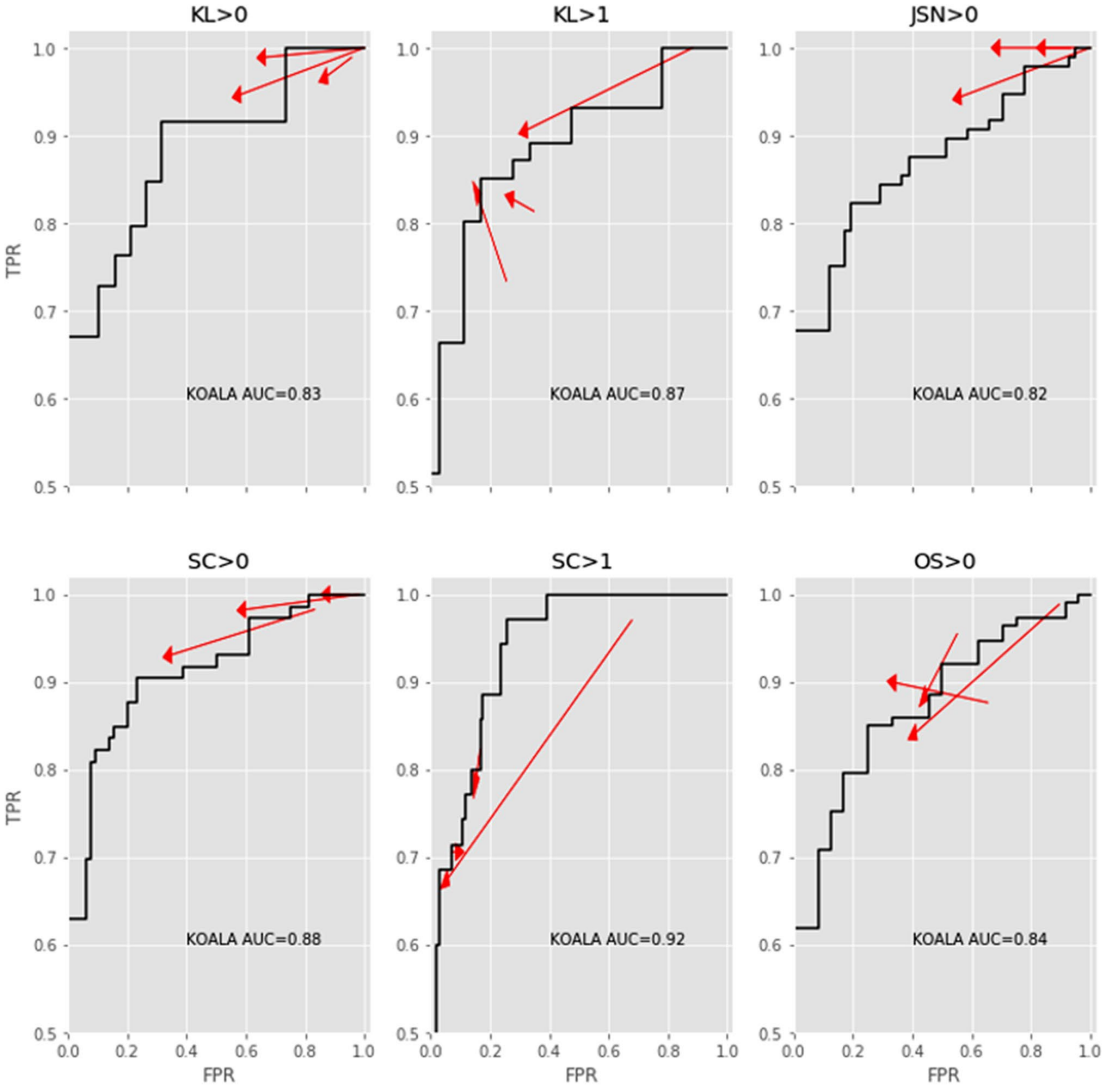
Changes to the true positive rate (y-axis, TPR) and false-positive rate (*x*-axis, FPR) for each individual senior reader for KL > 0, KL > 1, JSN > 0, sclerosis OARSI grade > 0, sclerosis OARSI grade > 1 and osteophyte OARSI grade > 0. The black line denotes the ROC curve for the AI software within the dataset. Arrows point from the unaided to aided modalities. Arrows pointing upward and left indicate absolute improvements in detection ability. Note that even though some arrows point downward and left, the improvement in FPR was greater than the loss in TPR, representing a net increase in accuracy. *KL* Kellgren–Lawrence, *JSN* joint space narrowing, *SC* sclerosis, *OS* osteophyte

The effect of the AI software on senior reader performance was comparable to that of our previous findings for junior readers only [19]. A reduction in FPR was observed, with no or little effect on TPR. Notably, two readers showed simultaneously increased TPR and reduced FPR regarding the feature “presence of OA” (i.e. KL > 1). For the “presence of osteophytes”, increased TPR and decreased FPR was found for another reader (Fig. 4).

## Discussion

The main finding of the study was improvement in the senior reader agreement rate with aided analysis for KL grade, diagnosis of OA, JSN, osteophytes and sclerosis assessed on knee X-rays. Furthermore, the specificity and accuracy for all features mentioned increased with the AI-aided modality. Notably, the agreement and accuracy rates achieved when using aided analysis were comparable between senior readers and junior readers (from a different study).

Although over 100 commercially available AI applications similar to the tools investigated herein using CNNs are currently on the market, peer-reviewed literature on the impact of these systems on clinicians is scarce [18].

Nevertheless, reliable and homogenous evaluation of radiological images is necessary to improve the care of knee OA patients by means of timely treatment planning [29]. This applies particularly to the early stages of knee OA, in which well-established radiographic assessment scores such as the KL scale are prone to imprecision, as the incipient tissue damage characteristic of early OA is barely visible on X-rays [30, 31]. However, in settings with both junior and senior readers [32], as well as senior readers alone [29], assessment differences regarding the severity of radiographic knee OA features are found. Therefore, an AI-based tool supporting readers in decision-making may aid in the standardization of image evaluation.

In the current study, agreement rates for senior readers increased between 1.37-fold (for JSN) and 3.33-fold (for OA diagnosis) when utilizing AI software-aided labelling compared with unaided labelling. These observations are in line with a previous study on junior readers alone [19]. Comparable to previous results from our group on junior readers [19], use of AI-enhanced X-rays appears to standardize knee OA assessment among senior readers. This is of particular importance considering that initial agreement rates between senior readers and the OAI ground truth were evidently lower than those found between junior readers and the OAI ground truth, especially regarding OA diagnosis and KL grade. This discrepancy may be explained by the fact that orthopaedic specialists rely on their long-term experience when evaluating images rather than adhering to given scoring system definitions, as enforced during the truthing of the OAI. In the field of musculoskeletal radiology, comparable observations have been made by Peterlein et al*.* regarding developmental dysplasia of the hip assessment by ultrasonography [33], with similar performance found between medical students and paediatric orthopaedic surgeons [33]. Notably, in the present study, the junior and senior readers achieved similar agreement rates with AI software-aided labelling. This implies that orthopaedic specialists may benefit to a greater extent from AI software than senior residents. One may argue that any improvement in agreement with aided labelling may be related to some kind of cognitive bias—or “anchoring effect” [34], a phenomenon first observed in psychophysics [35]. It describes the situation in which a person’s decision is influenced to a considerable degree by a single, potentially irrelevant piece of information, i.e. the “anchor” [36]. As already outlined in our previous study [19], some facts eventually contradict this assumption. On the one hand, the readers had been specifically trained in assessment of X-ray features, and objective decision-making can be expected. On the other hand, improvements with aided labelling against the OAI consensus as the ground truth were mainly caused by an increase in specificity, reducing FPR (and thus overdiagnosis) with respect to the OAI consensus. Furthermore, the senior readers achieved better overall results regarding TPR and FPR with aided labelling compared to AI software alone. This implies a rather subordinate role of the “anchoring effect”, as the senior readers should have achieved results similar to the AI software unless they eminently relied on provided annotations.

As the pool of readers in the current study consisted of three board-certified orthopaedic surgeons, one may argue that generalizability was impaired. To overcome this issue, a specific ICC was calculated including the readers as random effects. Consequently, the pool of readers was treated as a sample of a larger pool, enabling better generalization of the results obtained.

Limitations of the study include the drop-out rate of 7.3% and the limited sample size of the 115 images ultimately analyzed. Furthermore, the number of readers involved might have biased the results obtained. Another potential source of bias was the 2-week interval chosen between the two assessments, which might have led to “memory bias” [37, 38]. Nevertheless, the effect of this bias is controversial in the literature, with studies on the presence of both strong [38] and weak [37] “memory bias” in imaging studies at two time points.

Moreover, AI might have been overfitted to the OAI dataset, eventually leading to a less pronounced difference between the senior and junior readers for varying datasets due to lower performance of the AI. Additionally, it cannot be ruled out that AI software itself sometimes classifies X-rays incorrectly, and aided image analysis would present readers with inaccurate information. Furthermore, the discrepant finding of better performance for senior residents compared to board-certified orthopaedic surgeons can only be explained by the hypothesis that experienced readers tend to analyze images in a less-structured and faster manner, relying on their long-term experience, but that less experienced readers are more likely to adhere to presented image classification systems.

In conclusion, use of AI-based KOALA software leads to improvement in the radiological judgement of senior orthopaedic surgeons with regard to X-ray features indicative of knee OA and KL grade, as measured by the agreement rate and overall accuracy in comparison to the ground truth. Moreover, the agreement and accuracy rates of senior readers were comparable to those of junior readers with aided analysis. Consequently, standard of care may be improved by the additional application of AI-based software in the radiological evaluation of knee OA.
